# Probabilistic nucleation governs time, amount, and location of mineral precipitation and geometry evolution in the porous medium

**DOI:** 10.1038/s41598-021-95237-7

**Published:** 2021-08-12

**Authors:** Mohammad Nooraiepour, Mohammad Masoudi, Helge Hellevang

**Affiliations:** CO2 Storage Research Group, Department of Geosciences, University of Oslo, Blindern, P.O. Box 1047, 0316 Oslo, Norway

**Keywords:** Environmental sciences, Geochemistry, Hydrogeology, Carbon capture and storage

## Abstract

One important unresolved question in reactive transport is how pore-scale processes can be upscaled and how predictions can be made on the mutual effect of chemical processes and fluid flow in the porous medium. It is paramount to predict the location of mineral precipitation besides their amount for understanding the fate of transport properties. However, current models and simulation approaches fail to predict precisely where crystals will nucleate and grow in the spatiotemporal domain. We present a new mathematical model for probabilistic mineral nucleation and precipitation. A Lattice Boltzmann implementation of the two-dimensional mineral surface was developed to evaluate geometry evolution when probabilistic nucleation criterion is incorporated. To provide high-resolution surface information on mineral precipitation, growth, and distribution, we conducted a total of 27 calcium carbonate synthesis experiments in the laboratory. The results indicate that nucleation events as precursors determine the location and timing of crystal precipitation. It is shown that reaction rate has primary control over covering the substrate with nuclei and, subsequently, solid-phase accumulation. The work provides insight into the spatiotemporal evolution of porous media by suggesting probabilistic and deterministic domains for studying reactive transport processes. We indicate in which length- and time-scales it is essential to incorporate probabilistic nucleation for valid predictions.

## Introduction

Heat flow, fluid flow, rock deformation, and chemical reactions resulting from temperature and pressure gradients, geomechanical stresses, and chemical disequilibria are manifested in coupled thermo-hydro-mechanical-chemical (THMC) processes governing the long-term behavior of the fluid-rock system in geo-environments^1,2^. Mineral nucleation and growth is a prime example where (geo)chemical reactions give rise to geometry evolution in porous media^3^. When precipitation reactions are ample, crystal accumulations can dramatically reduce porosity (amount of void space)^4,5^. Alteration in pore volume, in turn, changes the connectivity of the pore space^3^. Alterations in porosity and connectivity lead to pore space morphology changes, affecting properties such as tortuosity and permeability of the altered medium, and therefore, changes in fluid flow and solute transport^6–8^. Mineral precipitation may partially or entirely clog pores and throats in the porous medium and change pore size distribution^4,9,10^. Even small amounts of solid accumulation may render the pore structure impermeable^3^. Additionally, precipitation reshapes the available surface area for growth, leading to changes in the system’s reactivity, reaction progress, and reaction rates^6,10^.

In pristine natural systems, reactions tend to be slow and predictive, with gradual changes towards lower free energy. On the other hand, sudden natural events or human engineering may give rise to sharp THMC gradients resulting in rapid and, in many cases, non-predictive alterations^11–13^. For example, during geological carbon storage, CO_2_ pressure increases far above average. It acidifies the reservoirs with carbonic acid, leading to the accelerated dissolution of primary minerals, and precipitation of stable carbonate minerals^12,14–17^. Subsurface CO_2_ storage may also cause rapid salt crystal accumulations in the near-wellbore region, prompting pore-space clogging, reduced injectivity, and pressure build-up^4,5,9,18,19^. Similarly, geothermal heat and mass exchange perturbation cause rapid dissolution–precipitation reactions, scale formation, and operational challenges^1,20^. In nuclear waste disposal sites, geochemical interactions between the cement and clay fractions may trigger precipitation of secondary minerals such as impermeable calcite layers, enhancing blockage of solute transport^21–23^. A similar phenomenon may occur in mine waste deposits, where alteration rims and secondary mineral formation can restrict the reactivity, acid generation, and metal release over time due to surface passivation^24,25^. During treatment of contaminated groundwater, nucleation and precipitation of secondary solids cause substrate passivation (often carbonates) and limited buffering capacity^26^. In all of the mentioned examples and many others, strong feedback loops exist among the mineral reactions and solid formation on the one hand, and fluid flow and solute transport on the other hand.

Understanding and predicting porous media evolution in natural settings has far-reaching impacts beyond the engineered and human-made processes and systems. In natural environments, chemical, flow, and transport properties can vary notably over different time- and length-scales^3,12,27,28^. Predictions of such complex natural or engineered perturbations require numerical modeling, which is far from trivial. Knowledge of the individual mineral reactivity, representative mathematical equations for the thermodynamics and mineral kinetics, and numerical models are needed to solve the strongly non-linear systems of combined flow, transport, and reactions at spatiotemporal scales ranging from individual pores to entire sedimentary basins. The reactive transport modeling (RTM) framework must satisfy the specific system of interest, ranging from advective–diffusive transport of solutes close to a mineral surface^10,13,16,29,30^ to fluid flow and reactions at the field- or regional-scales and over geological time^11,31–33^.

However, intrinsic complexities of the spatial and temporal evolution of parameters describing porous media’s composition and structure are simplified in RTM by several assumptions and interdependencies describing transport and reactivity. Reactive transport processes are routinely modeled using averaged upscaled parameters within a representative elementary volume (REV). There are relationships and interdependencies between porosity, reactivity, evolving surface area, and transport properties. These relationships are primarily empirical and derived either by curve fitting to laboratory datasets or theoretical models. Imperfectly described properties and processes, heterogeneities, and complexities of mineral-fluid-porous medium systems, in reality, pose a challenge to delineate chemical perturbations and predict system evolution.

Mineral reaction rates are traditionally computed using the Transition State Theory (TST) developed about four decades ago^34,35^. The straightforward nature of the TST equation has made it, by far, the most used kinetic model employed to predict a range of natural and engineered phenomena^36–40^. Today, the TST model is implemented in most commercial reactive transport packages. However, using the equation to predict crystal growth rates violates the principal assumptions made to derive the TST model. Moreover, the TST approach’s simplification and assumptions lead to unrealistically high precipitation rates, even at low supersaturation levels^11,12^. This knowledge led to new kinetic models and formulations based on classical nucleation theory (CNT)^11,41,42^. Because of the complex interactions between reaction processes and transport across time- and length-scales, even when the total amount of secondary precipitates is precisely captured, it is far from trivial to predict the evolution of transport properties. Apart from flow and transport, the kinetic models fail to predict where crystals will nucleate and form in the pore space. It is essential to predict the location and distribution of the precipitates besides their amount, particularly when clogging is expected^3^. Nucleation is the pre-growth process that controls the primary position of any mineral precipitation. Mineral nucleation is a probabilistic process where crystals nucleate anywhere given similar conditions such as surface properties, supersaturation, and temperature. It is imperative to use a probabilistic approach or an upscaled physically sound representation to understand the effect of mineral precipitation on porous medium hydrodynamics.

We present a mathematical formulation of a probabilistic model for mineral nucleation for the first time to our knowledge. As a pore-scale realization of probabilistic nucleation, we developed a Lattice Boltzmann Method (LBM) reactive transport model to simulate crystal nucleation and precipitation on a two-dimensional mineral surface substrate. Furthermore, we conducted sensitivity analysis to study the impact of reaction rate constant and nucleation rate on the distribution of precipitated minerals, and consequently, geometry evolution of the system. Additionally, 27 microfluidic experiments (3 × 3 × 3 sets of supersaturation–temperature–time) were conducted on the surface of a heterogeneous geomaterial substrate to visualize mineral nucleation and precipitation, study the dynamic of growth, and facilitate comparison between the laboratory and numerical outcomes.

As variations in the porous medium’s properties are intimately linked to the spatial distribution of the secondary precipitates, we quantify the evolution of experimental and numerical modeled systems at different physiochemical conditions via mapping the Shannon Entropy of the spatial mineral distributions across time. The resulting entropy curves describing the stochastic process can be used to decipher the spatiotemporal evolution of the system. We further present a conceptual framework based on probabilistic nucleation to delineate solid formation for various times and system sizes. Probabilistic and deterministic domains were introduced, describing the spatial and temporal scale over which the precipitate renders the porous medium heterogeneous. Finally, we propose a preliminary upscaling approach for continuum-scale studies and highlight where it is crucial to incorporate probabilistic criterion for reactive transport modeling.

## Results and discussion

### Effect of supersaturation and experimental time

**Figure 1:**
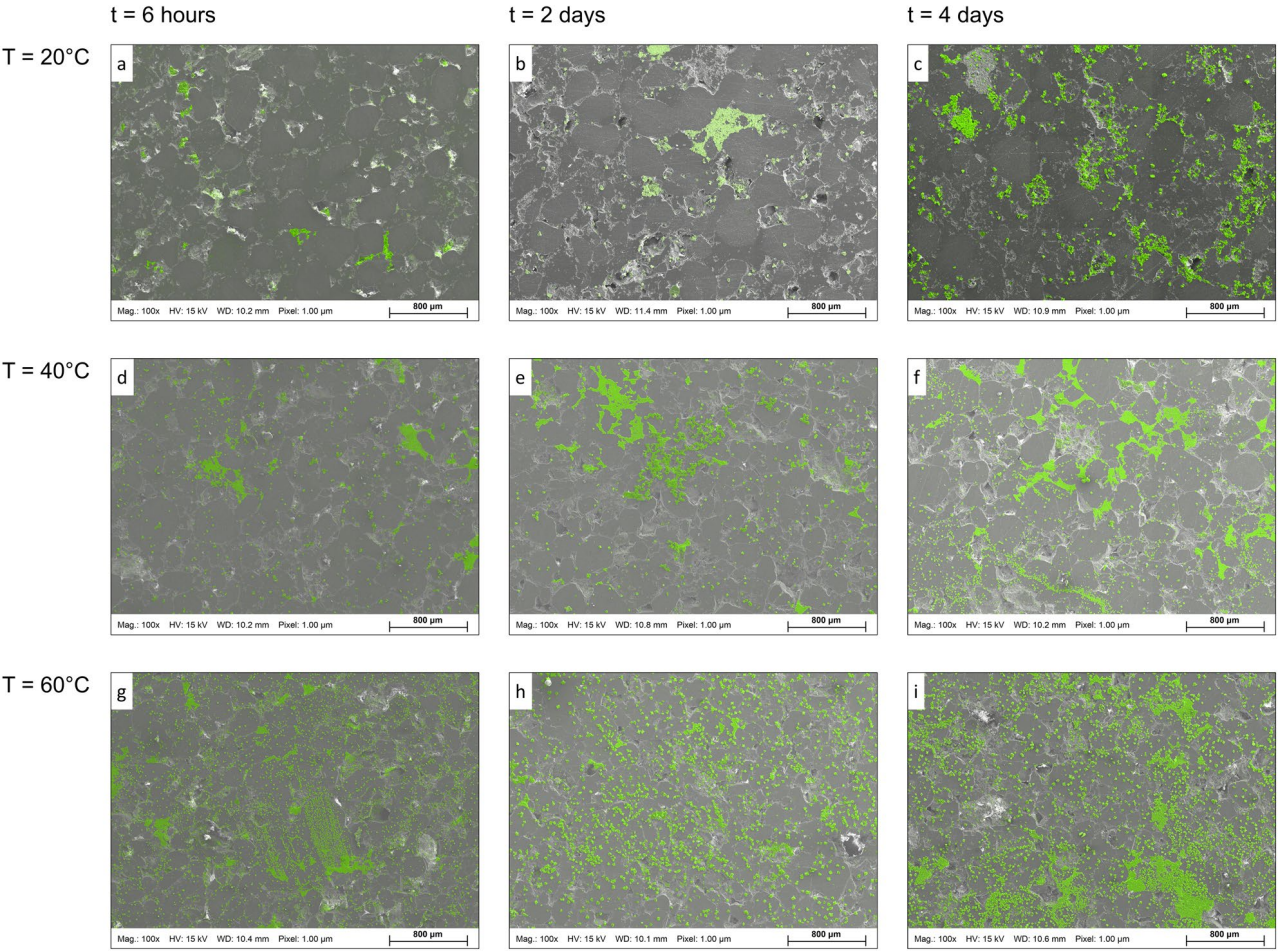
Mosaic maps of calcium carbonate crystal precipitation on the surface of natural quartz-rich sandstone substrates at different temperatures (T) and elapsed times (t) for the 130 × supersaturation experimental set. The mosaic maps demonstrate the superimposed energy-dispersive X-ray spectroscopy (EDS) to identify precipitated crystals (color-coded in green) on top of the backscatter scanning electron microscopy (SEM) micrographs. Each mosaic map covers a 10.5 mm^2^ area, with individual pixels representing a 1 µm^2^ surface area.

Classical nucleation theory tells us that supersaturation, temperature, and interfacial free energy are the main factors determining the number of stable growing crystals. We carried out nine sets of laboratory experiments at three supersaturation levels (Ω = Q/K = 15, 50, and 130 ×) and three temperatures (T = 20, 40, and 60 °C) with three different experimental elapsed time intervals (t = 6 h, 2 days, and 4 days). Figure 1 shows the mosaic maps of sandstone substrates at different temperatures (T) and times (t) for the 130 × supersaturation experimental set. The mosaic maps demonstrate the superimposed energy-dispersive X-ray spectroscopy (EDS) analysis to identify calcite crystals (color-coded in green) on top of the backscatter SEM micrographs. Two trends are evident in Fig. 1. First, an increase in T and t caused a rise in the number of crystals and the total area covered by precipitated calcite. The size of patches (connected precipitates) also shows an increase at higher T–t conditions (e.g., Fig. 1i).

**Figure 2:**
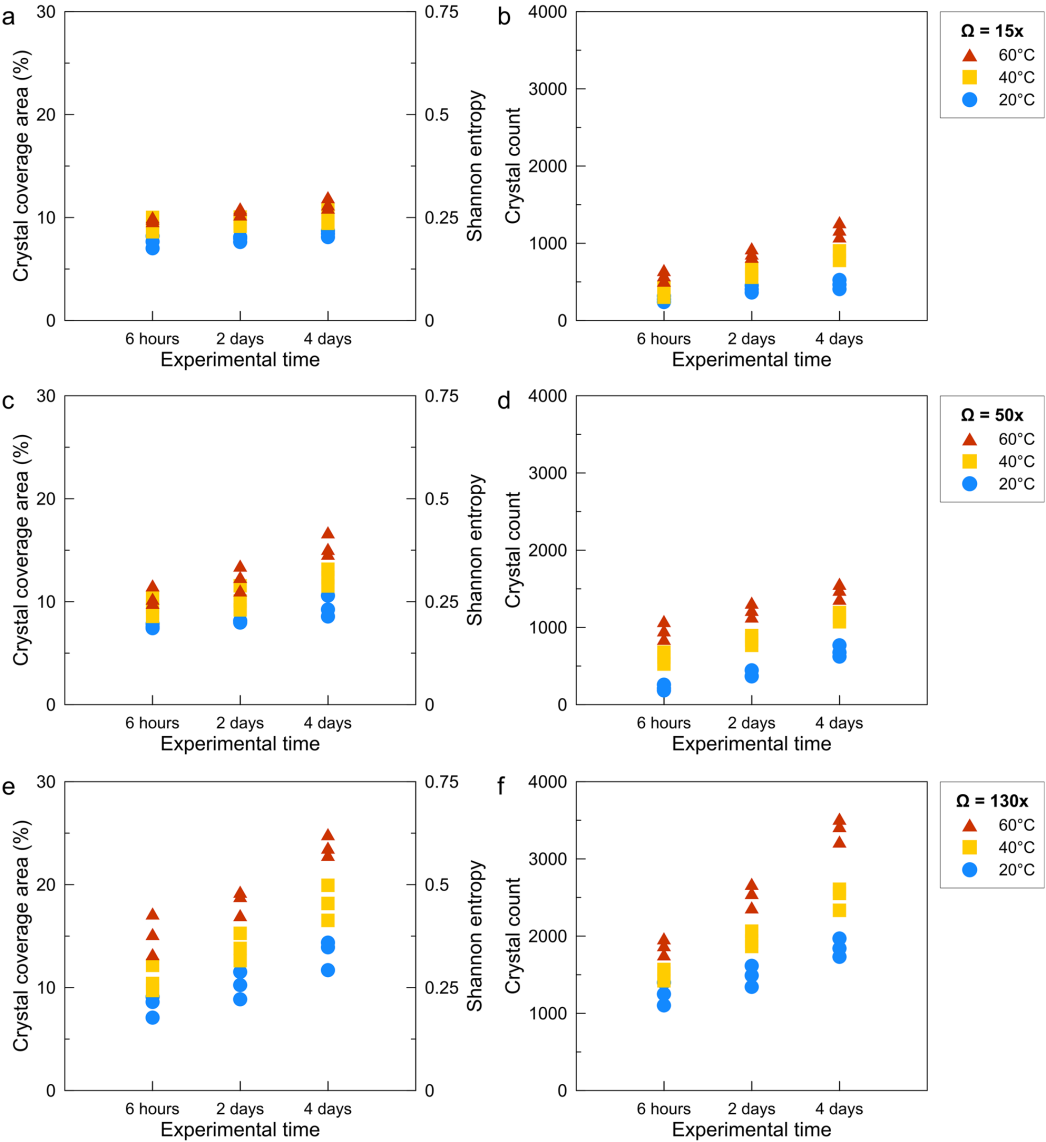
Total coverage area (%), computed Shannon entropy, and the number of the precipitated crystals plotted against the experimental sampling time. (top) 15 × (middle) 50 ×, and (bottom) 130 × supersaturation experiments as a function of testing temperatures. The results are derived from digital image processing of three random locations within each mosaic map.

The image processing results of 27 experiments (3 × 3 × 3 sets of Ω–T–t) are presented in Fig. 2, where total coverage area (%) and crystal count are plotted versus elapsed time. Figure 2 also shows the computed entropy of each mosaic map in the second ordinate (y-axis) to measure randomness within the system. For each experiment, three random locations of 10.5 mm^2^ areal coverage are mapped.

With the increase of supersaturation, moving from top to bottom in Fig. 2, the distinction between the data points representing different t and T becomes more visible. The crystals’ coverage area and count also increased remarkably for higher Ω values, particularly for Ω = 130. The increase in the coverage area and crystal counts for a given Ω are subtle for T = 20 °C compared to elevated T. Additionally, at low supersaturation (Ω = 15), the number of new crystals after initial sampling (t = 6 h) shows a limited increase for the rest of the experiments (t = 2 and 4 days) for all three T sets. However, at higher supersaturations (Ω = 50, 130), enough solute is present in the experimental solution to support the continued nucleation and growth of calcite crystals. As a result, a semi-exponential increase in area and count over time is discernible in Fig. 2.

### Effect of reaction rate, nucleation rate, and simulation time

The experiments were performed on a heterogeneous substrate, but because of the probabilistic nature of nucleation, the spatial–temporal evolution of crystal nucleation and growth will also vary on homogenous substrates. To underline the importance of incorporating the probabilistic nucleation model, we chose to simulate a homogenous solid surface for better representation without adding complexities of heterogeneous substrates into the results. We developed a Lattice Boltzmann Method (LBM) reactive transport model to investigate the effect of reaction rate constant (k_G_) [mol/m^2^/s] and nucleation rate (R_N_) [nuclei/s] on the extent, distribution, and mineral precipitation patterns on a homogenous surface. We modeled nine scenarios to cover a wide range of physicochemical conditions (here, the reaction rate constant and nucleation rate). The simulation scenarios include reaction rate constant (k_G_) over three orders of magnitude (10^–7^ to 10^–5^) and nucleation rate (R_N_) over five orders of magnitude (10^–4^ to 10^0^). Figure 4a provides a better overview of the entire simulation scenarios.

**Figure 3:**
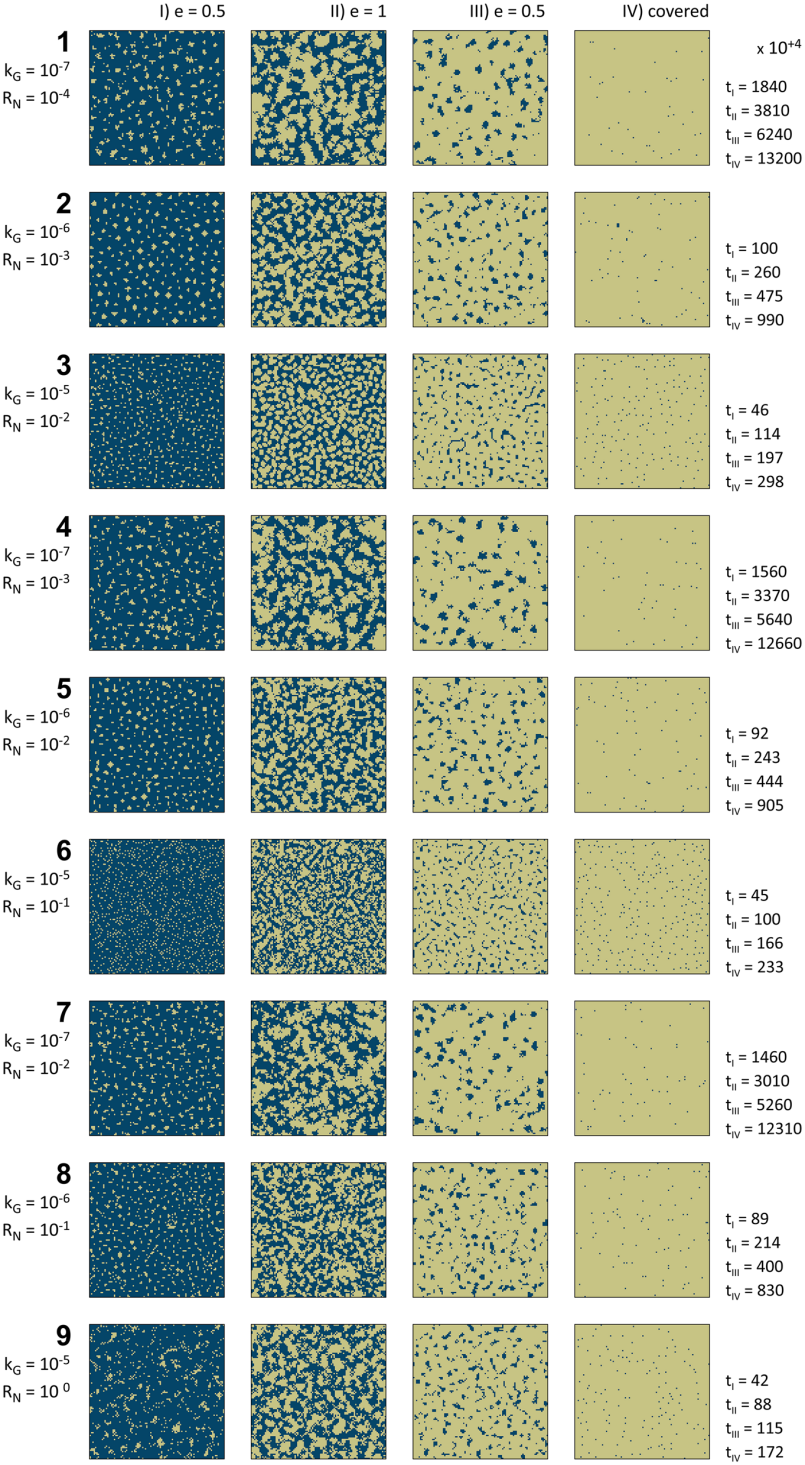
Precipitation maps of nine Lattice Boltzmann Method (LBM) reactive transport simulation scenarios implementing probabilistic nucleation model are given over the entire evolution time of the system. Each map represents a 400 × 400 µm system with a grid resolution of 4 µm. For each scenario, simulation number, reaction rate constant (k_G_), nucleation rate (R_N_), entropy level (e) together with the corresponding simulation time-steps (× 10^4^) are shown. The simulation scenarios represent reaction rate constant (k_G_) over three orders of magnitude (10^–7^ to 10^–5^), and nucleation rate (R_N_) over 5 five orders of magnitude (10^–4^ to 1^0^).

Figure 3 shows the precipitation maps for nine LBM simulation scenarios over the evolution time (4 µm resolution in a 400 × 400 µm system), their k_G_ and R_N_ values, along with the time-steps for each subfigure. For each scenario, we show four maps representing crystal precipitation during the system’s geometry evolution at four entropy levels (0.5 upward, 1, 0.5 downward, and when substrates become fully covered and entropy reaches a constant value).

**Figure 4:**
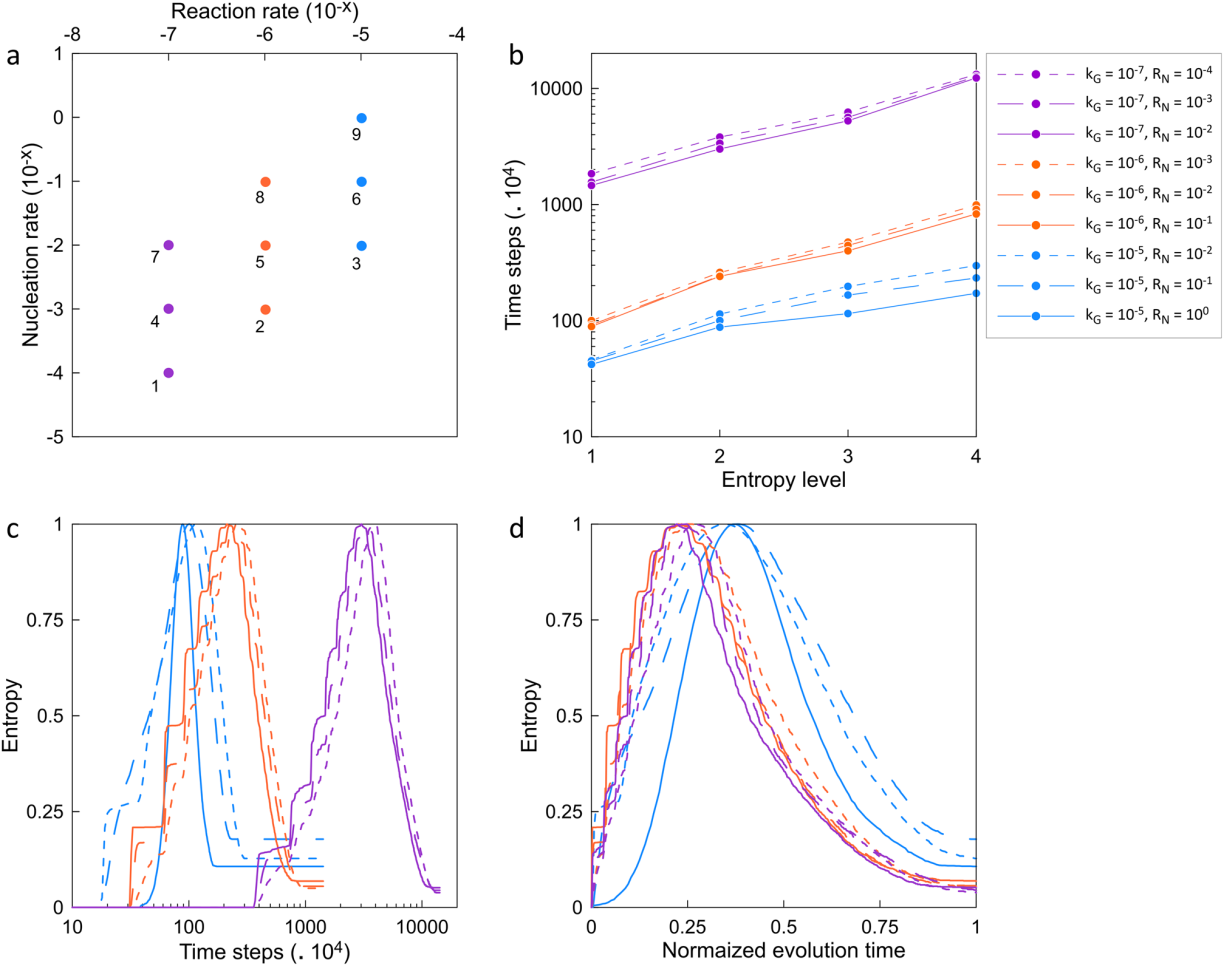
Analysis of Lattice Boltzmann Method (LBM) simulation results with probabilistic nucleation model at different physiochemical conditions. (**a**) Overview of reaction rates constant (k_G_) and nucleation rate (R_N_) for simulation scenarios; (**b**) simulation time (× 10^4^) versus entropy levels for precipitation maps in Fig. 3; (**c**) computed Shannon entropy during simulation time (× 10^4^) for subplots in Fig. 3 until solid accumulations entirely covers the substrate; and (**d**) computed Shannon entropy versus normalized evolution time, where the horizontal axis is the normalized simulation time with respect to the overall time span for each graph in part c. Scenarios with similar color represent similar reaction rates constant (k_G_), and different line styles demonstrate different nucleation rates (R_N_). At each k_G_, three orders of magnitude R_N_ was studied.

In Fig. 3, once solid precipitation fully occupies a grid, it grows due to the continuous supply of supersaturated solution and continuous nucleation on the secondary (precipitated crystals) and primary substrate. The simulation scenarios indicate that k_G_ has primary control over covering the substrate with nuclei and subsequently crystal accumulation (Fig. 3). For example, for the same R_N_ = 10^–2^ in cases 3, 5 and 7 with different k_G_ (10^–5^ to 10^–7^), a significant difference in the evolution time (crystal precipitation covering the substrate’s surface) is noticeable as it is also graphically depicted in Fig. 4b. Moreover, k_G_ governs the interconnection and patchy accumulation of nuclei and thus crystals, which may lead to controlling the occupation of pore space during reactive transport processes. The pore occupancy and interconnection behavior make the underlying basis for defining a probabilistic clogging behavior caused by crystal nucleation and growth. Although the nine scenarios and their subplots in Fig. 3 may seem graphically analogous, there are several principal differences among them. Besides precipitation time (or simulation time), the comparison between the scenarios with different k_G_ indicates that lower k_G_ scenarios facilitated (a) bigger crystal accumulations, (b) bigger inter-crystal pore volumes in early stages of solid formation, particularly in subplots I and II, and (c) better surface coverage and lower inter-crystal porosities in the final stage.

### Quantification of spatiotemporal variations

We quantified the degree of disorder within the simulated scenarios using the Shannon entropy of the spatial mineral distributions across time. The entropy defined in information theory captures increasing randomness within the system^43,44^. Fig. 4 depicts the analysis of different simulation scenarios presented in Fig. 3. Subplots of Fig. 4 are as follows (a) k_G_, R_N_ and simulation numbers; (b) the time that each scenario reaches a certain entropy level (0.5 upward, 1, 0.5 downward, and when solid precipitations entirely cover the surface and entropy reaches a constant value). The vertical axis in part “b” is in logarithmic-scale; (c) computed Shannon entropy during entire simulation time for subplots in Fig. 3 a constant value representing geometry evolution of the system. The horizontal axis in part “c” is in logarithmic-scale; and (d) computed entropy versus normalized evolution time, where the horizontal axis is the normalized simulation time with respect to the time span for each graph.

Figure 4a shows the reaction and nucleation rates of the conducted LBM simulations. The similarity in rates is depicted for various scenarios. As Fig. 4b demonstrates, the simulation cases can be categorized based on their k_G_ values (similar colors). The lower the k_G_, the longer the evolution time of the system. The marked difference is particularly notable for k_G_ = 10^–7^.

Figure 4c represents the Shannon entropy for each growth map per time step to assess the degree of randomness associated with the probabilistic of nucleation and growth. For a given simulation scenario, randomness and disorder show a Gauss–Laplace distribution. Entropy starts from a fully ordered system with no solid precipitation on the substrate. It increases as the solid phase starts emerging on the surface until randomness reaches the maximum value (1). Afterward, the overall disorder declines as more and more surface areas are covered, and eventually, entropy approaches zero towards an ordered state (fully covered). The entropy plots show a Gaussian bell function (normal distribution) with varying variance (σ^2^). One also can argue that the q-Gaussian distribution might better represent the collective plots with varying q and β parameters. The q-Gaussian is a generalization of the Gaussian in the same way that Tsallis entropy is a generalization of standard Boltzmann–Gibbs entropy or Shannon entropy. In three scenarios (3, 6 and 9 with k_G_ = 10^–5^) in which fast precipitation is observed, the entropy curves show a right skewness with a rather abrupt increase in the degree of disorder since the first crystal. The faster the growth, the narrower the standard deviation of the Gaussian bell. In Fig. 4c, the logarithmic scale in the horizontal axis helps distinguish three different sets of evolution pathways grouped by similarity in reaction rates. However, the normalized entropy curves (Fig. 4d) demonstrate comparable q-Gaussian distributions for different simulation cases. The similarity is more preannounced for the slower simulation sets with k_G_ = 10^–6^ and 10^–7^. A stepwise evolution path is also observable for these two sets, particularly during the disorder increase half plot (left side of the Gauss–Laplacian PDFs in Fig. 4d).

As solute concentration in the LBM simulations is defined to maintain the growth, one may hypothesize that the ratio between the reaction rate and nucleation rate solely controls the growth pattern. Thus, it can be used as a proxy for predicting porous medium’s evolution across scales. However, the simulation results refute the hypothesis as scenarios with similar ratios demonstrate dissimilar growth maps for the extent, distribution, and precipitation patterns of the solid phases (Figs. 3 and 4). Such deviation occurs because of the increased affinity to nucleate and grow adjacent or on top of secondary substrates. As experimental results show, the affinity for nucleation and growth of secondary minerals is higher adjacent or on top of the newly formed crystals than the original foreign substrate (Fig. 1). It incorporates another layer of complexity into the system, which is necessary to take into account to capture the reality of nucleation and crystal growth. This dimension is realized and integrated into the probabilistic nucleation model (refer to the Methods Section and the Supplementary Information) and the described LBM reactive transport model. The formation of the first nucleus on the substrate creates a secondary substrate with a higher potential (probability) to form the following nuclei. In other words, the marked difference between the interfacial free energy of the primary substrate and the secondary substrate (precipitated nuclei) determines the probability and affinity of new nucleation events.

### System geometry evolution in time and space

**Figure 5:**
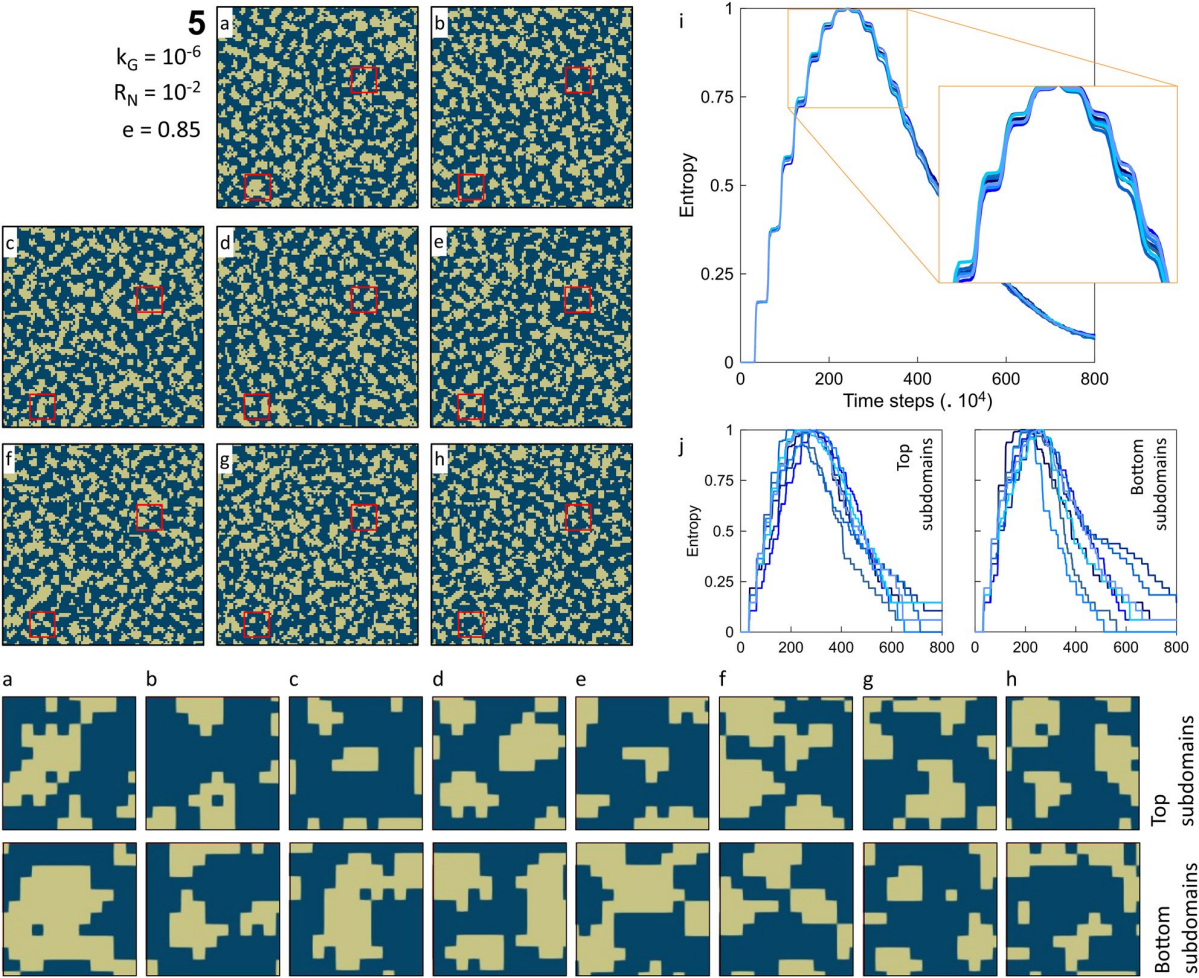
(Left, **a**–**h**) eight different realizations of simulation scenario number 5 (k_G_ = 10^–6^, R_N_ = 10^–2^ at e = 0.85) indicating stochastic amount and distribution of surface solid precipitation as implemented in probabilistic nucleation and crystal growth model. The red squares show the crystal accumulation status for two random REV subdomains. The subdomains (top and bottom squares) are enlarged for better comparison for each realization. The simulation number, reaction rate constant (k_G_), nucleation rate (R_N_), and entropy level (e) are shown. (right, **i**) the computed Shannon entropy values for eight realizations during the entire simulation time (× 10^4^), describing the system’s evolution pathways. (right, **j**) the computed Shannon entropy for subdomains (top and bottom squares) during the entire simulation time (× 10^4^).

In the probabilistic reactive LBM simulations, the randomness of the spatial distributions is ensured via stochastic parameters controlling location and number of nuclei. Figure 5a–h represents eight different realizations for simulation scenario 5 with R_N_ = 10^–2^ and k_G_ = 10^–6^ at 0.85 entropy level (at 180 × 10^4^ time steps) to investigate and compare the randomness of the crystals’ spatial distribution. After the initial placement of nuclei, they start to grow until they occupy a grid and become visible on the growth maps. Having access to solute concentration, solid phases grow and occupy adjacent grids, providing an extended secondary substrate for the subsequent nucleation events. The chance of nucleation on the secondary substrates is more than the primary substrates. This interplay between the reaction and nucleation following a probabilistic algorithm governs the precipitation patterns (Fig. 5). The process resumes until crystals cover the substrate entirely. As shown in Fig. 5i, the overall evolution described by the entropy for all the realizations is similar. Although, the pattern of surface precipitations, crystal locations, and their amount is different. The difference in realizations in Fig. 5 is more evident if we consider smaller subdomains (red squares) in each realization.

In Fig. 5, two subdomains are randomly selected for each realization, namely top and bottom subdomains. The surface area of subdomains is 50 times smaller than the original realizations. Each subdomain is enlarged to evaluate variations in smaller system sizes visually. For these two randomly picked locations, different precipitation amounts, different precipitation locations, different growth patterns are observed. Moreover, the probabilistic control on mineral nucleation and precipitation within these two subdomains is better discernible than the original domains in Fig. 5. To quantify the spatial randomness of distributions, we computed entropy for each subdomain in Fig. 5j. The degree of scatter and dissimilarity in evolution paths is notably higher than in Fig. 5i, particularly for the bottom subdomain’s entropy plot. At a similar evolution time (at 180 × 10^4^ time steps), the original realizations showed a 0.85 entropy level while the disorder level in subdomains varies from 0.65 to 0.95 (Fig. 5j). It highlights that the size of the system and time matters for the entropy and thereby the randomness of the spatial distribution.

Comparing the experimental results (Figs. 1, 2) and the simulation outcomes (Figs. 3, 4) indicates that laboratory surface precipitation maps in Fig. 1 are on the first half of the system evolution. This system can be categorized as a system with a slower reaction where probabilistic nucleation is of great importance for modeling its behavior. Figures 3 and 4c,d show that different systems take similar evolution paths but at different time-scales. Each evolution path starts with an ordered system, and after the formation of nuclei, mineral growth starts to happen, and the system goes toward a maximum disorder (entropy of 1). The degree of disorder decreases until all the surface is covered with the precipitates. All the systems follow this Gaussian path. We call this path the “probabilistic window” because, as shown in Fig. 5, a system can go down this path in countless ways. After the probabilistic region, the system goes to a deterministic region. We call the boundary between these two regions the transition zone.

It is worth mentioning that spatial randomness of the nucleation and growth phenomenon can be further evaluated when besides the Shannon entropy, multivariate distributions of surface co-occurrences be quantified to capture the disorder in spatial distribution and relative precipitation patterns^45^. It can provide necessary insights for proposing a clogging model and predicting changes in transport properties (porosity–permeability).

### Scaling reactive transport phenomena

The reactive transport field lies at the crossroad of several disciplines in Earth and Environmental Sciences such as geochemistry, hydrogeology, hydrology, geology, and fluid mechanics. Describing the behavior of geological systems affected by natural processes and human-made activities is a challenging task. The challenges originate from intrinsic complex properties of minerals, solutions, porous media, along with imperfectly characterized heterogeneities and anisotropies of Earth materials. In addition, the reactive transport field features multiscale systems and processes in time and length, ranging from nanoseconds to millions of years in time and nanometers to planetary scales in size. Such broad spatiotemporal domains represent systems with ultra-slow to extremely fast evolutions. Therefore, it is crucial to use up/down-scaling concepts and representative models to connect different time and length scales to predict where and when the processes occur within the system.

The fluid-rock interactions are good examples of multiscale processes. While advection and diffusion occur at the pore-scale level, it is in most cases represented by Darcy-scale, where porous media is considered a continuum. Therefore, in addition to paying attention to individual processes at their time- and length-scales, one must devise a framework to appropriately upscale or downscale different processes with different spatiotemporal resolutions. Down/upscaling is necessary for studying how various scales interact and how they join forces in overall coupled processes. In many instances, insights from the observation scale may have limited applicability to surface or subsurface systems. One famous example is the discrepancies between laboratory and field mineral reaction rates, which have been the topic of numerous literature contributions in geochemistry^33,46^. Across these time and length extremes, mathematical equations and constitutive laws require modifications, or as Steefel^33^ coined the term, mathematical and numerical models need to become “scale aware”. Further complications in the transient and heterogeneous settings may occur because of coupling between several reactive transport processes or events at Earth’s surface or subsurface systems. Such couplings or events can cause “hot moments” with extensive impacts on the system, even though they may represent a limited percentage of the total time frame or system size^47^.

**Figure 6:**
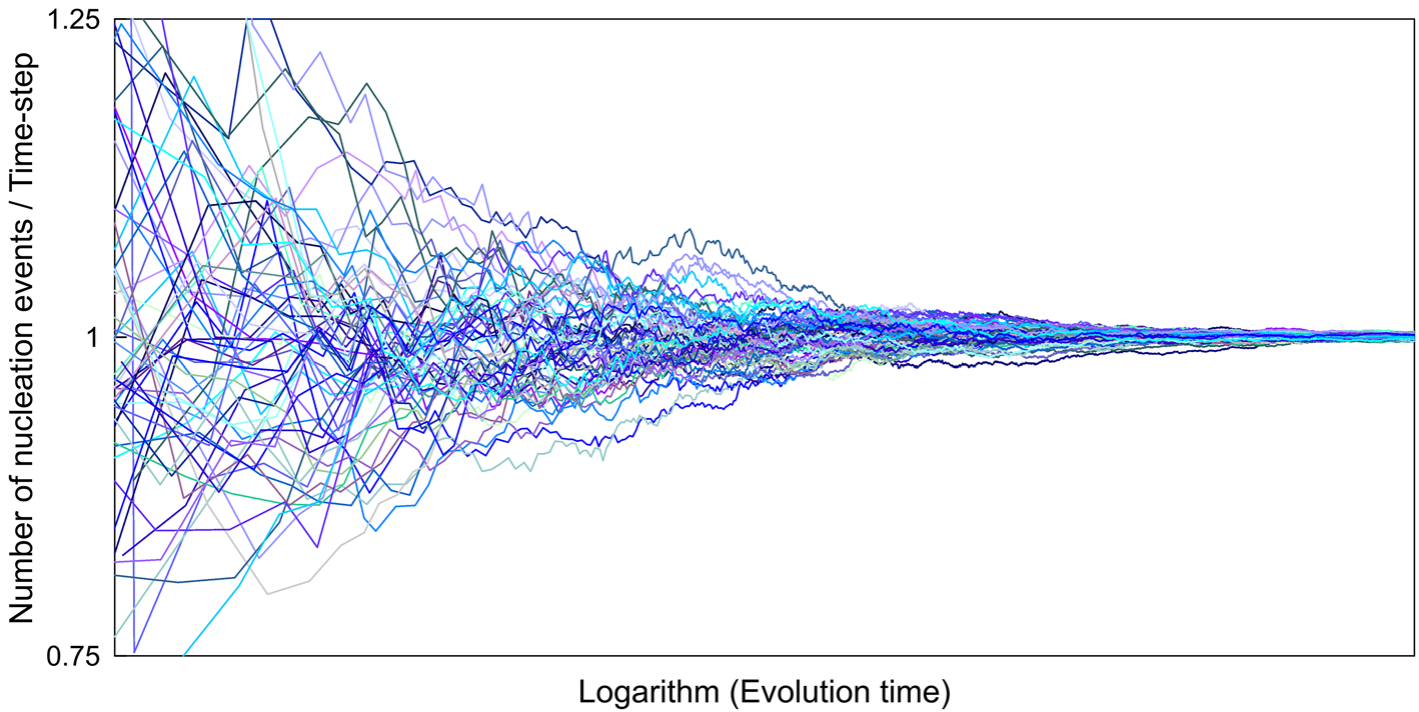
The number of nucleation events per time step versus the logarithm of the system’s evolution time based on the probabilistic nucleation theory. It shows numerous stochastic scenarios, their development dynamics, and convergence as the evolution time increases.

In the current work, the nucleation event is the so-called hot moment, the occurrence of which determines the system’s future dynamics^48^. Nucleation events, a probabilistic process by nature, governs the location and timing of crystal precipitation. We suggest that one can reduce such (apparent) discrepancies by implementing probabilistic and deterministic domains for studying reactive transport processes across time- and length-scales (system physical size). In order to make the “probabilistic window” clearer, let us contemplate the nucleation process only. We run the probabilistic nucleation model (refer to the Methods Section and the Supplementary Information) numerous times for a defined system. Figure 6 shows the cumulative number of nucleation events divided by the number of time steps versus the system’s time evolution (cumulative number of time steps) following the probabilistic nucleation theory. It portrays the overall status of the nucleation events across the evolution time of the system for a given induction time. The stochastic simulation results represented in Fig. 6 demonstrate that each individual’s realization follows a random evolution over time. We can see the same behavior as shown in Fig. 4c and d. After the first nucleation event, the number and timing of subsequent nucleation events are probabilistic, changing over a probability distribution domain. As the system evolves, different realizations of the given simulation scenario converge, and they tend to unity.

Nucleation is indeed a probabilistic process that controls subsequent crystal precipitation. However, for the longer time-scales when the system evolves to a certain degree, a deterministic proxy can describe the nucleation event without losing generality or violating crystal growth physics. Induction time, in turn, determines the boundary between the probabilistic and deterministic domain (transition zone) and how fast it transforms from a disordered stochastic system to one that crystal nucleation can be handled and predicted deterministically. The induction time depends on the type and physiochemical condition of the reaction. Following the classical nucleation theory (CNT) formulation, interfacial free energy between the substrate and nucleating phase determines the type of the reaction, and saturation ratio and temperature define the physiochemical condition.

**Figure 7:**
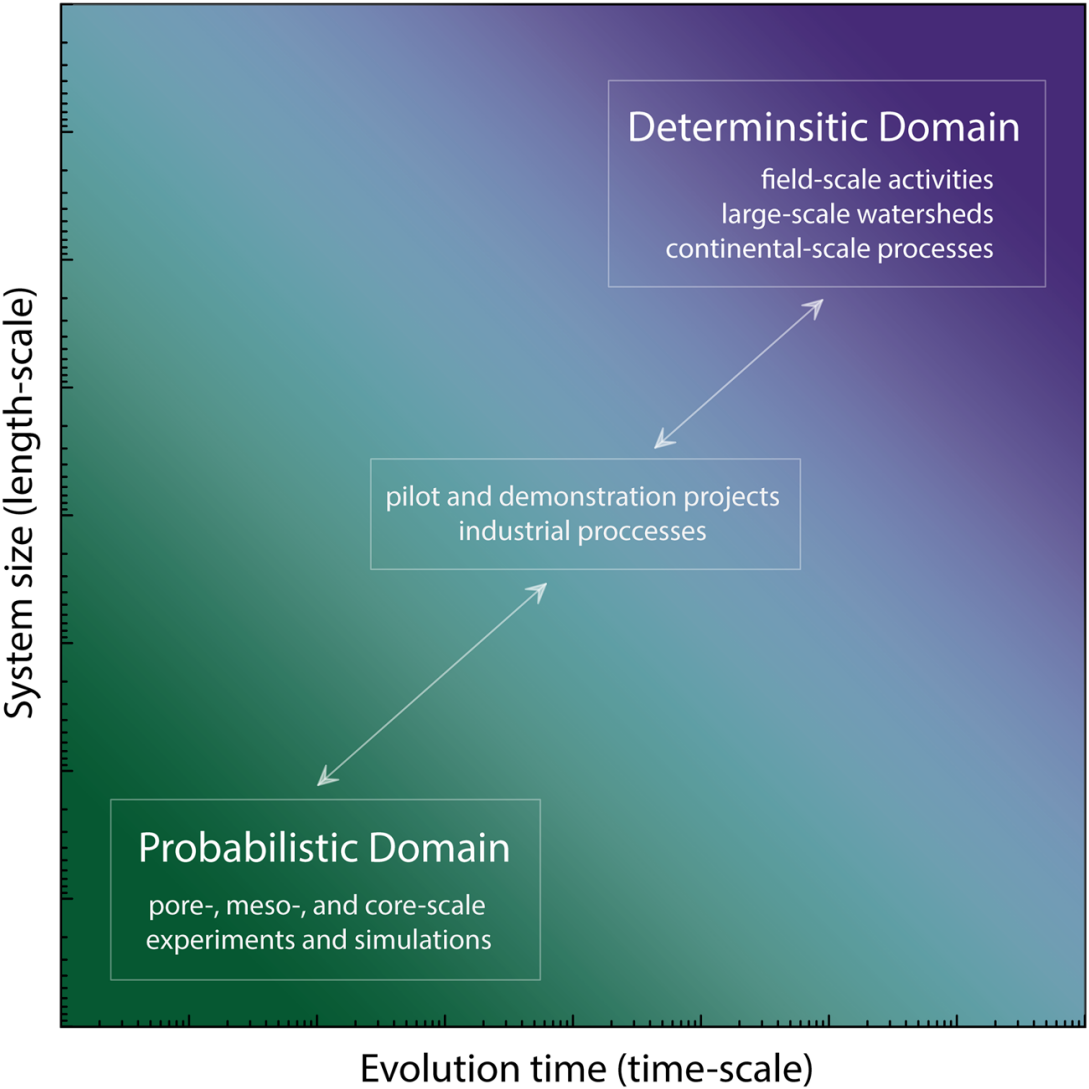
A conceptual framework specifying probailistic and determisntic domains to predict mineral nucleation for various spatiotemporal scales. The induction time determines the boundary between the probabilistic and deterministic domain (transition zone).

Figure 7 shows the conceptual framework we propose to handle and predict mineral nucleation for various spatiotemporal resolutions. In Fig. 7, the horizontal axis represents system evolution (time-scale), and the vertical axis indicates system size (length-scale). The vertical axis can also be a proxy for available surface area for the occurrence of precipitation events. The proposed framework consists of two domains, namely, probabilistic and deterministic domains. The probabilistic model better represents slow processes, small-scale systems, and extended induction times. A deterministic model can describe fast processes and large-scale systems. For example, in Fig. 4 if we evaluate the subdomains (red squares), it characterizes with more probabilistic behavior than the entire domain. At a given time instance, the subdomains might be entirely occupied or with minimal amounts of precipitates.

A reactive system may exhibit a continuous behavior from entirely probabilistic to nearly deterministic. Pore-scale, meso-scale and core-scale experimental and numerical studies fall into the probabilistic domain. Field-scale activities, large-scale watersheds, and continental-scale are placed in the deterministic domain. Processes and systems that share characteristics of both domains in terms of intermedial length- and time-scales are located in the transitory realm. Pilot projects and the majority of industrial processes are two examples of the transitory realm. To make sure that simulations in larger scales consider all the physics behind a particular phenomenon, the results of any simulations (or experiments) in the probabilistic domain should be upscaled to the larger domains. Porosity–permeability relations used in continuum-scale modeling to account for precipitation/dissolution are examples of this upscaling between two domains. However, none of the current porosity–permeability relations consider and honor the probabilistic nucleation.

It should be noted that time is a relative concept in Fig. 7. The time interval is entirely different for different reactions and different physiochemical conditions. The interface between the two domains is controlled by induction time and is a function of temperature, supersaturation, and interfacial free energy. As induction time for a given process changes, it moves the boundary between the domains and determines how fast or slow it will enter the deterministic domain. For extended times and large volumes, the continuum variable in the deterministic domain undergoes slight changes considering the spatiotemporal size. However, in the probabilistic domain below a specific time span and volume, marked variations are expected as dimensions approach individual pore scales. As we showed in Fig. 5 for each realization, even within the probabilistic domain, the bigger the system, the more even overall variations in the defined REV.

For separation between the deterministic and probabilistic domains (Fig. 7), one will get a mean value and a certain spread around the average value at any point along the progress (Fig. 6), and one may define the limit to be at a given variation around the mean. In other words, the deterministic domain can start even if there is not yet a unique value for all runs in Fig. 6, since a small spread will still provide the right outcome for the deterministic simulations, and thus, choosing to operate in the deterministic domain for reactive transport modeling (RTM). One consideration and research question for upscaling is to determine the allowed spread around the mean value in the deterministic domain.

Besides microscopic or microfluidic surface studies (Figs. 1 and 3), column experiments that investigate secondary minerals’ precipitation during the flow of a reactive fluid need to implement considerations of probabilistic nucleation^49^. The authors documented that during the flow of CO_2_-acidified brine through a column packed with basaltic glass, only a few but large patches of carbonates were formed in random locations along the column. Hellevang et al.^49^ claimed that laboratory observations could only be explained by total control of the nucleation process. Hence, it can only be simulated with a reactive transport model with a probabilistic nucleation approach. The calcium carbonate synthesis experiments presented in this study (Fig. 1) provide similar insights as we showed a stochastic behavior for the location and precipitation pattern of the calcite crystals.

### Implementing probabilistic nucleation model into pore- and continuum-scale numerical simulations

This section set forth a workflow to describe the geometry evolution of porous medium during reactive transport as a result of mineral precipitation. The proposed probabilistic nucleation model (refer to the Methods Section and the Supplementary Information) can be described as a “scale aware” model because it honors and incorporates downscaling and upscaling. The probabilistic nucleation model defines the induction time for the unit of surface area, i.e., ${\tau }_{p}$[m^2^ s nuclei^−1^]. Therefore, for the scale-dependent induction time to form, the probabilistic induction time (being a constant value for a reference unit area) needs to be divided by the available surface area for nucleation. i.e., ${\overline{\tau }}_{p}={\tau }_{p}/{A}_{n}$. The bigger the grid resolution, the bigger the substrate and area available for nucleation, and consequently, the smaller the scale-dependent induction time:

1 $$\underset{{A}_{n}\to \infty }{\mathrm{lim}}\frac{{\tau }_{p}}{{A}_{n}}=\underset{{A}_{n}\to \infty }{\mathrm{lim}}{\overline{\tau }}_{p}=0$$

New nucleation events are more likely to occur with shorter induction times as a nucleus forms if the following inequality satisfies:

2 $$\Delta t\ge {\overline{\tau }}_{p}$$

Furthermore, when the time scale increases, the elapsed time (Δt) increases accordingly. As a result, the chances of new nucleation events increase. Additionally, a larger grid size is often accompanied by a larger time step, and larger time steps typically correspond to larger grid sizes. With this interplay between grid size and time step, the RTM scenario may reach a point where the probabilistic model is no longer required to be implemented. The nucleation can be handled via the traditional deterministic approach in those cases. However, it should be highlighted that, even for the cases that fall into the deterministic domains, the nucleation phenomenon is still probabilistic. The only difference is that the probability of nucleation in such a large grid system with a long time step from a stochastic perspective is high enough that incorporating a probabilistic model might not be relevant anymore.

In the laboratory results (Figs. 1 and 2) and LBM simulations (Figs. 3 and 4), we indicated that supersaturation and reaction rate strongly control the mineral nucleation and crystal growth and the morphology of the newly precipitated solids. Altering pore network characteristics, including connectivity, tortuosity and architecture, as well as changes in surface roughness induced by solid formation, can render into numerous distinct variations in absolute and effective permeability. Such reality may not be captured by Kozeny–Carman, Hagen–Poiseuille, Fair–Hatch type or power-law porosity–permeability relationships, which are implemented in almost all available reactive transport packages. The question is how the proposed probabilistic nucleation and precipitation model may assist us in constructing a more predictive and precise reactive transport model. To explain this, we categorize the reactive transport model representation of the porous medium into pore-scale and continuum-scale. In pore-scale models (non-continuum scales), one can physically simulate the actual geometry of the porous medium and hence directly study the precipitation pattern. However, in the continuum-scale, the parameters describing the fluids’ and the porous medium’s properties are described with average values within a representative elementary volume (REV).

In the pore-scale simulations, it is essential to implement the proposed probabilistic nucleation model for capturing the location of nucleation events and the consequent precipitation pattern for a given process or system. The pore-scale results can then be upscaled to provide insights into the larger scale models via developing porosity–permeability relationships or clogging models. It should be noted that the clogging models might be a significant source of uncertainty in modeling mineral precipitation^50^ because different precipitation processes change the porous medium’s geometry in their specific ways. As a result, a similar change in porosity can lead to entirely different alterations in permeability depending on the process causing the changes, the host environment and materials, and the mechanism behind the changes. In our opinion, a single macroscopic relationship can hardly provide reliable insights for pore-size-dependent reactions, scale-controlled (time and size) reactions, and scenarios where small changes in porosity lead to significant changes in permeability or vice versa.

In continuum-scale, pore morphologies are not pertinent, and the alterations in hydrodynamics of the porous medium are modeled via clogging models (porosity–permeability relationships). The process-specific clogging model should be developed via pore-scale simulations with the probabilistic nucleation approach. In other words, the precipitation pattern is handled by upscaling the results of pore-scale modeling. If continuum-scale simulation scenario falls into the probabilistic domain, the probabilistic nucleation model is essential in answering the two following questions. First, whether any nucleation will occur in a grid cell or not. It is particularly influential for smaller length scales, modeling scenarios in which minerals show extended induction time, and reactions with slow kinetics. In contrast to the deterministic approach that specifies nucleation in all the grids with certain saturation ratio levels, the probabilistic nucleation model points out that nucleation events may not occur within all the grids. Second, the frequency of nucleation events in each grid cell. The frequency of stable nuclei, in turn, controls the amount of solid precipitation in a grid. If continuum-scale simulation scenario falls into the deterministic domain ($\Delta t\gg {\overline{\tau }}_{p}$), the averaged hydrodynamic properties can be predicted, disregarding the impact of nucleation events on mineral precipitation and growth. Simply put, the routine deterministic workflow in RTM can be followed.

The selection of the crystal growth model does not necessarily need to be sophisticated. However, it is paramount to be capable of predicting rates based on the available reactive surface area S_G_ (provided from the stable nuclei) and thermodynamic driving forces such as Ω. The nucleation rate can be upscaled the same way as the reaction rate. One also may consider scaling up according to the type of the minerals within the grid or interfacial free energy. Tracking the number of secondary particles and their crystal size distribution is highly valuable later when down-scaling to understand crystal growth’s effect on rock properties such as permeability and mechanical strength.

## Methods

### Calcium carbonate synthesis experiments

Stock solutions of calcium chloride (CaCl_2_) and sodium bicarbonate (NaHCO_3_) were prepared by adding the solid salt crystals of a certain weight to the deionized water (Milli-Q water), as presented in Table 1. We used the PHREEQC v.3 package^51^ for aqueous geochemical calculations before the experiments to compute solute supersaturation. The supersaturation (Ω) corresponds to the saturation ratio, being the ion activity product divided on the equilibrium constant.

**Table 1:** Supersaturation and molality of stock solutions prepared for calcium carbonate synthesis experiments.

	Supersaturation^a^	Molality of dissolved salts	
		Sodium bicarbonate	Calcium chloride
Solution A	15 (15.85)	0.005	0.0005
Solution B	50 (47.86)	0.007	0.001
Solution C	130 (131.83)	0.01	0.002

^a^*Ω* = *Q/K*, where Q is the ion activity product and *K* the equilibrium constant for the reaction.

A natural quartz-rich sandstone (Brumunddal sandstone) was selected as the substrate. The Brumunddal red sandstones were deposited under desert conditions at the beginning of the Permian period^52^. Cylinders of 2.5 cm long and 1.5 cm in diameter were cut from a core sample and then polished in several stages to prepare 27 geomaterial substrates.

Nine sets of experiments were carried out at three supersaturation levels (Ω = 15, 50, and 130 ×) and three temperatures (T = 20, 40, and 60 °C). First, two stock solutions corresponding to a given supersaturation were carefully added to the test vessel to prepare the 250 ml experimental solution. Then three geomaterial substrates were placed and fully submerged inside the test vessel. Next, the test vessel was sealed and placed inside a temperature-controlled air bath. A forced convection benchtop oven ensured temperature uniformity and airflow during the tests. We sampled out a reacted substrate for each experiment set after 6 h, 2 days, and finally 4 days of experimental time (t).

### Surface characterization and mineral identification

Scanning electron microscopy (SEM) with backscatter electron imaging (BEI) and energy-dispersive X-ray spectroscopy (EDS) were used to study the surface structure of the tested substrates, element mapping, and qualitative mineral identification. A Hitachi SU5000 FE-SEM (Schottky FEG) provided the SEM analyses, and the EDS was performed by a Dual Bruker XFlash system and a high-resolution automated electron backscatter diffraction (HR EBDS) system. Carbon coating of substrates was carried out to improve imaging quality, increase chemical analysis precision, and better topographic examination while avoiding surface charging and potential thermal damages. For each substrate (representing different Ω, T, and t), three random locations were analyzed, and a mosaic map of nine ordinary SEM images (3 × 3) was acquired at each locality. Each mosaic map covers a 3786 × 2781 µm region, with each pixel representing 1 µm^2^ surface area.

### Digital image processing

We followed a workflow for digital image processing of surface mosaic maps to quantify precipitated calcium carbonate crystals on top of the sandstone substrate. The superimposed calcium phase map (color-coded in green) on top of the substrates’ mosaic map was selected as an input. First, several filters were applied to reduce noises and enhance contrast in each input map. The mosaic map was then converted to a binary image after thresholding colors. The inverted (mask) transformation of the binary map was analyzed in ImageJ/Fiji open-access image processing package^53^. Finally, precipitated calcium carbonate crystals were identified, outlined, and quantified to describe their geometrical characteristics.

### Probabilistic nucleation model

Nucleation rate (R_N_) [nuclei s^−1^] as the number of nucleation events on a defined surface per time unit is defined as:

3 $${R}_{N}=\frac{{A}_{N}}{\tau }$$

where A_N_ [m^2^] is the available area for nucleation and τ [m^2^·s.nuclei^−1^] is the induction time, by definition the elapsed time from the onset of starting a nucleation event by establishing a constant supersaturation and detecting a stable growing nucleus (crystal). In this work, induction time varies statistically and is considered a random variable in the probabilistic nucleation model. The probabilistic induction time (τ_P_) is stochastically spread around the deterministic (measured or reported) induction time (τ_N_), being the mean of the distribution.

In this work, the deterministic mean induction time (τ_N_) is computed based on the classical nucleation theory (CNT):

4 $$\mathrm{ln}\left({\tau }_{N}\right)=\frac{\widehat{\Gamma }{\sigma }^{3}}{{T}^{3}{\left(\mathrm{ln\Omega }\right)}^{2}}-\mathrm{ln}\left({k}_{N}\right)$$

where T [K] is absolute temperature, Ω is saturation ratio providing the thermodynamic driving force for nucleation, σ [J m^−2^] is interfacial free energy between the nucleating phase and the substrate, and k_N_ [nuclei m^−2^ s^−1^] is nucleation rate constant. $\hat{\Gamma }$ is a lumped parameter expressed as:

5 $$\widehat{\Gamma }={\beta \nu }^{2}{\mathrm{K}}_{B}^{-3}$$

where β is a geometry factor, υ [m^3^ molecule^−1^] is the molecular volume of the nucleating phase, and k_B_ is the Boltzmann constant, 1.38 × 10^−23^ [J K^−1^].

To calculate τ_N_, σ and k_N_ values need to be determined from laboratory experiments, in which nucleation rates are measured at different supersaturation levels. The nucleation rates are then incorporated into the CNT to obtain σ and k_N_. Additionally, the geometry factor (β) is a function of the nucleus shape. For instance, for a spherical nucleus, β is considered equal to 16π/3 for homogeneous nucleation, but for heterogeneous nucleation, it is no longer 16π/3.

In the probabilistic nucleation model, the probability of forming a nucleus during a time step is expressed by a Gauss–Laplace (normal) probability density function (PDF). The following equations give the probability density function, P(x), and cumulative distribution function, F(x), of the Gaussian distribution:

6 $$P\left(x\right)=\frac{1}{sd\sqrt{2\pi }}{e}^{-\frac{{\left(x-m\right)}^{2}}{2{sd}^{2}}}; 0\leq x\leq 2{\tau }_{N}$$

7 $$F{^{\prime}}\left(x\right)=P\left(x\right)$$

where, m is the mean (m = τ_N_), and sd is the standard deviation (sd = τ_N_/4) of the random variable x. To find probabilistic induction time (τ_p_), a random number with probability (p) of 0 < p < 1 is generated, then τ_p_ (between 0 and 2τ_N_) is found such that the value of the normalized F(x) at τ_p_ is equal to the p. Briefly, F(x = τ_p_) = p. Assigning a value to τ_p_ is iterated in each time step of the reactive transport simulation.

It is assumed that one stable nucleus forms for each induction time that is shorter than the period that the solution’s saturation ratio remains unchanged or increased in contact with the substrate. Therefore, if we call the random number generator N times to find ${\overline{\tau_{p}}}$ from F(x) until $\mathop \sum \limits_{{N}}{\overline{\tau_{p} }}>\Delta t$, N-1 stable nuclei will form.where, Δt is the time interval during which crystal counts are computed. The number of precipitated crystals in each time step provides the reactive area provided for the crystal growth. The reactive area is used as an input parameter for the growth rate computation.

Although the PDF describing the induction times might not necessarily be Gaussian, assuming a Gauss–Laplace distribution for τ_p_ provides a good enough approximation. According to the Central Limit Theorem, in a population with mean μ, standard deviation σ, and sufficiently large random samples from the population with replacement, the sample means are approximately normally distributed. It holds regardless of the source population being normal or skewed, given that the sample size is sufficiently large (~ n > 30).

The Python script handling pore-scale probabilistic nucleation model is provided as Supplementary Information for open-access usage to assist researchers studying reactive transport processes in the straightforward implementation of the code and incorporating probabilistic nucleation and precipitation into the RTM simulations.

### LBM reactive transport model

We used the Lattice Boltzmann Method (LBM) to solve the advection–diffusion-reaction equation for tracking the concentration of different species:

8 $$\frac{{\partial C}_{j}}{\partial t}+\nabla .\left(-{D}_{j}{\nabla C}_{j}+u{C}_{j}\right)={R}_{j}$$

where ${\mathrm{C}}_{\mathrm{j}}$[NL^−3^] is the aqueous concentration of species j, ${\mathrm{D}}_{\mathrm{j}}$[L^2^T^−1^] is the diffusion coefficient of species j in water, u is velocity [LT^−1^], and ${\mathrm{R}}_{\mathrm{j}}$[NL^−3^ T^−1^] is the source or sink term due to reactions for species j. The discretized LB equation used to solve the advection−diffusion–reaction equation (the mass transport) is as follows:

9 $${g}_{i}^{j}\left(x+{c}_{i}\Delta t, t+\Delta t\right)={g}_{i}^{j}\left(x, t\right)-\frac{\Delta t}{{\tau }_{g}}\left[{g}_{i}^{j}\left(x, t\right)-{g}_{i}^{eq, j}\left(x, t\right)\right]+\Delta t{Q}_{i}^{j}\left(x, t\right)$$

10 $${g}_{i}^{eq, j}={w}_{i}{C}^{j}\left(1+\frac{{c}_{i}.u}{{c}_{s}^{2}}\right)$$

In which ${\mathrm{g}}_{\mathrm{i}}^{\mathrm{j}}$ is discrete distribution function, ${\mathrm{g}}_{\mathrm{i}}^{\mathrm{eq},\mathrm{j}}$ is equilibrium distribution function, and C^j^ is concentration of species j. Δt is time resolutions, τ_g_ is relaxation time, ${\mathrm{c}}_{\mathrm{s}}$ is lattice speed of sound, and c_i_ and w_i_ are discrete velocity sets and weighting coefficients.

The D2Q9 lattice scheme used in the present study, where 2 is the number of spatial dimensions (x and y) and 9 is the number of discrete velocities, w_0_ = 4/9, w_1–4_ = 1/9, and w_5–8_ = 1/36 and

11 $${c}_{i}=\left\{\begin{array}{c}\left(\mathrm{0,0}\right), i=0\\ \left(\mathrm{cos}{\theta }_{i}, \mathrm{sin}{\theta }_{i}\right), {\theta }_{i}=(i-9)\frac{\pi }{2}, i=1-4\\ \left(\mathrm{cos}{\theta }_{i}, \mathrm{sin}{\theta }_{i}\right)\sqrt{2}, {\theta }_{i}=(2i-9)\frac{\pi }{4}, i=5-8\end{array}\right.$$

After solving Eq. (9), the concentration of species j is calculated as follows:

12 $${C}^{j}=\sum_{i=0}^{8}{g}_{i}^{j}$$

The source and sink term is given by:

13 $${Q}_{i}^{j}\left(x, t\right)={w}_{i}\left[{q}_{N}^{j}\left(x,t\right)+{q}_{G}^{j}\left(x,t\right)+{q}_{S}^{j}\left(x,t\right)\right]$$

14 $${q}_{N}^{j}=\frac{d{C}_{N}^{j}}{\Delta t}$$

15 $${q}_{G}^{j}=\frac{d{C}_{G}^{j}}{\Delta t}$$

16 $${q}_{S}^{j}={D}_{j}\left({SR}^{0}-SR\right), if \, SR<{SR}^{0}$$

The subscripts N, G, and S in Eq. (11) denote the nucleation, growth, and infinite source of the solution on top of the substrate.

The simulation domain is 400 × 400 μm with a grid resolution of 4 μm representing a homogeneous nonreactive substrate. The domain is initially oversaturated with solute mineral A (Ω_0_ = 60). The periodic boundary condition is applied for all the boundaries. An infinite source of a solution oversaturated with mineral A on top of the substrate was imposed. Simulating a two-dimensional (2D) system, the infinite source is implemented via a source term.

The first step in the LBM simulations is nucleation. Nucleation is the necessary condition for growth or for the reaction to start {A_(aq)_ ⇄ A_(s)_}. In this work, we control the necessary condition using the reactive surface area:

17 $${R}_{G}={k}_{G}S\left(1-\Omega \right)$$

In Eq. (15), R_G_ [mol s^−1^] is the growth or reaction rate, k_G_ [mol·m^-2^·s^-1^] is the growth rate constant, S [m^2^] is the reactive surface area, and SR is the saturation ratio:

18 $$SR=\frac{{C}_{A}}{{C}_{eq}}$$

where, C_A_ [mol L^−1^] is the concentration of mineral A in the aqueous phase, and C_eq_ [mol L^−1^] is the equilibrium aqueous concentration of mineral A.

The reaction surface area is equal to zero as long as there is no nucleation in a grid. When the first nucleation event occurs, it provides a reactive surface area, and if the saturation ratio is more than one, the nucleus starts to grow. The more the crystal grows, the more the reactive surface area is provided. We assume that:

- A nucleus provides an equivalent surface area as a sphere of 10 nm radius.
- The secondary crystal grows in the middle of a grid as a cube.
- A grid is regarded as a solid-filled grid when the solid phase occupies more than 75% of its volume (dx^3^).

Moreover, the nucleation and growth of the secondary phase on the initial substrate provide a new substrate in which the chance of nucleation will be higher. The weighted arithmetic mean based on the surface area, Eq. (17), is used to calculate the average surface energy when we have different substrates in a grid cell. In the present work, we assumed that the interfacial free energy between the nucleating phase and the initial substrate is 50 times larger than the interfacial free energy between the nucleating phase and the precipitated solids.

19 $$\tilde{\sigma }=\frac{{\sum }_{i}{S}_{i}{\sigma }_{i}}{{\sum }_{i}S_{i}}, i=substrate \, type$$

## Supplementary Information

Supplementary Information.

## Data Availability

The data and materials that support the findings of this study are available from the corresponding author upon reasonable request. The numerical code used in this study is available (with restrictions) upon reasonable request.
