# Supplementary material for: Probabilistic nucleation governs time, amount, and location of mineral precipitation and geometry evolution in the porous medium
Source: Sci Rep. 2021 Aug 12;11:16397. doi: 10.1038/s41598-021-95237-7 (PMC8361100; doi:10.1038/s41598-021-95237-7)
Supplement: Supplementary file 1 — Supplementary Information. [file 41598_2021_95237_MOESM1_ESM.pdf]

Supplementary Information

Probabilistic nucleation governs time, amount, and location of mineral precipitation and geometry evolution in the porous medium

Mohammad Nooraiepour*, Mohammad Masoudi and Helge Hellevang

CO_2 Storage Research Group, Department of Geosciences, University of Oslo
P.O. Box 1047 Blindern, 316 Oslo, Norway

*Corresponding author: Mohammad Nooraiepour, mohammad.nooraiepour@geo.uio.no

Python script for probabilistic nucleation model

This section provides the Python script to implement the *probabilistic nucleation model* into reactive transport models (RTM). Before incorporating the code, we advise carefully considering the mathematical representation of the probabilistic nucleation model as presented and discussed in the Methods Section of the paper.

Please do cite the main paper, as you utilize the model and the following script:

```python
def Probabilistic_nucleation_Halite(Sigma_init,SR,elapsed_time,S_A,solid_mol,S_init):
    # Sigma_init   = initial interfacial free energy between the nucleating phase and the initial substrate in each grid (before precipitation)
    # SR           = saturation ratio
    # elapsed_time = period during which the solution's saturation ratio remains unchanged or increased in contact with the substrate
    # S_A          = surface area provided by the neighbouring grids
    # solid_mol    = mole of precipitated minerals in each grid
    # S_init       = initial surface area (the surface area of the initial substrate in each grid)
    # mvol         = molar volume of the nucleating phase
    # sigma1       = initial interfacial free energy between the nucleating phase and the secondary substrate (nucleating phase itself)
    # kN           = nucleation rate constant
    # T            = absolute temperature (K)
    # gamma_hat    = a lumped parameter (refer to the paper, Equation 5)
    # f            = cumulative distribution function
    # crystal      = number of stable crystal in each grid
    # P            = a random number normally distributed between 0 and 1
    # vol_nucleus  = volume of one stable nucleus
    # dC_ph        = difference in concentration (physical unit)
    # dC           = difference in concentration (LB unit)
    # dx3          = grid volume


    S_crystal = (solid_mol*mvol)**(2/3) # S_crystal= dx_crystal**2, we assume that crystals are cubic (m)

    # nucleating surface area:
    S_av = S_A + S_init + (4*S_crystal) #new surface provided by the neighbours (S_A) + initial surface of the substrates - surface of one side of the percipitant + 5 sides of the cube
    # average interfacial free energy (Equation 17):
    sigma_av = (sigma1*S_A + (5*S_crystal) * sigma1 + (S_init - S_crystal) * Sigma_init)/S_av

    # classical nucleation theory (Equation 4 in the paper):
    sigma3=sigma_av**3
    ln_SR=np.log(SR)
    ln_SR2=ln_SR**2
    ln_kN=np.log(kN)
    ln_tau=((gamma_hat*sigma3)/(T3*ln_SR2))-ln_kN
    tau=np.exp(ln_tau) # Classic Nucleation Theory (CNT)
    # tau: induction time  (m2.s/#nuclei) therefore we need to divide it to the surface area:
    tau=tau/S_av

    # normal distribution parameters:
    sig =1.0  # standard deviation
    mu = 1.0  # mean
    # calculates theoretical cumulative normal distribution:
    x = np.arange(-3, 5, 0.005) # x-axis is transfered from (-3,5) to (0,2*tau)
    # it is required to find an step size and step_tau_p, which satisfy:
    # x = -3:0.005:5 is proportional to 0:step_tau_p:2*tau
    step_tau_p= (2.*tau-0)/((5-(-3))/0.005)
    f = (1 /(np.sqrt(2*np.pi*sig**2))) * np.exp(-((x - mu)**2)/(sig**2))
    f = f.cumsum()
```

```python
    f /= f[-1]

    crystal = np.zeros(S_A.shape)
    t_p = np.zeros(S_A.shape)

    while np.any (t_p < elapsed_time):
        P=np.random.uniform(0,1,[S_A.size,1])
        tau_p_position = np.argmin(np.abs(f - P), axis=1) # to find the position of tau_p so that f(x=tau_p)=P.
        t_p += (0 + tau_p_position*step_tau_p)
        # one stable nucleus forms for each t_p shorter than elapsed_time:
        crystal[t_p < elapsed_time] = crystal[t_p < elapsed_time] + 1

    # updates other parameters:
    solid_mol += vol_nucleus*crystal/(mvol) # (mol)
    dC_ph = (vol_nucleus*crystal/(mvol))/(dx3) # [mol/m3]
    dC = dC_ph * (dx3)/Conv_mol # LB unit


    return crystal, solid_mol,dC
```
